# Super-Enhancer-Associated Long Non-Coding RNA LINC01485 Promotes Osteogenic Differentiation of Human Bone Marrow Mesenchymal Stem Cells by Regulating MiR-619-5p/RUNX2 Axis

**DOI:** 10.3389/fendo.2022.846154

**Published:** 2022-05-19

**Authors:** Wenli Gu, Xiao Jiang, Wei Wang, Prabhakar Mujagond, Jingpeng Liu, Zhaoyi Mai, Hai Tang, Simin li, Hui Xiao, Jianjiang Zhao

**Affiliations:** ^1^ Stomatological Hospital, Southern Medical University, Guangzhou, China; ^2^ Regional Centre for Biotechnology, Faridabad, India; ^3^ Shenzhen Stomatological Hospital, Southern Medical University, Shenzhen, China

**Keywords:** LINC01485, miR-619-5p, RUNX2, super-enhancers, bone marrow mesenchymal stem cells, osteogenesis, osteogenic differentiation

## Abstract

**Objective:**

To investigate the mechanisms of super-enhancer-associated LINC01485/miR-619-5p/RUNX2 signaling axis involvement in osteogenic differentiation of human bone marrow mesenchymal stem cells (hBMSCs).

**Methods:**

Osteogenic differentiation of hBMSCs was induced *in vitro*. The expression levels of LINC01485 and miR-619-5p during osteogenesis were measured using quantitative real-time polymerase chain reaction (qRT-PCR). Osteogenic differentiation was examined by qRT-PCR, western blot, alkaline phosphatase (ALP) staining, ALP activity measurement, and Alizarin Red S (ARS) staining assays. Thereafter, the effects of LINC01485 and miR-619-5p on osteogenic differentiation of hBMSCs were evaluated by performing loss- and gain-of-function experiments. Subsequently, a fluorescence *in situ* hybridization (FISH) assay was employed to determine the cellular localization of LINC01485. Bioinformatics analysis, RNA antisense purification (RAP) assay, and dual-luciferase reporter assays were conducted to analyze the interactions of LINC01485, miR-619-5p, and RUNX2. Rescue experiments were performed to further delineate the role of the competitive endogenous RNA (ceRNA) signaling axis consisting of LINC01485/miR-619-5p/RUNX2 in osteogenic differentiation of hBMSCs.

**Results:**

The expression of LINC01485 was up-regulated during osteogenic differentiation of hBMSCs. The overexpression of LINC01485 promoted osteogenic differentiation of hBMSCs by up-regulating the expression of osteogenesis-related genes [e.g., runt-related transcription factor 2 (RUNX2), osterix (OSX), collagen type 1 alpha 1 (COL1A1), osteocalcin (OCN), and osteopontin (OPN)], and increasing the activity of ALP. ALP staining and ARS staining were also found to be increased upon overexpression of LINC01485. The opposing results were obtained upon LINC01485 interference in hBMSCs. miR-619-5p was found to inhibit osteogenic differentiation. FISH assay displayed that LINC01485 was mainly localized in the cytoplasm. RAP assay results showed that LINC01485 bound to miR-619-5p, and dual-luciferase reporter assay verified that LINC01485 bound to miR-619-5p, while miR-619-5p and RUNX2 bound to each other. Rescue experiments illustrated that LINC01485 could promote osteogenesis by increasing RUNX2 expression by sponging miR-619-5p.

**Conclusion:**

LINC01485 could influence RUNX2 expression by acting as a ceRNA of miR-619-5p, thereby promoting osteogenic differentiation of hBMSCs. The LINC01485/miR-619-5p/RUNX2 axis might comprise a novel target in the bone tissue engineering field.

## Introduction

Critical-sized bone defects in oral and maxillofacial regions occurring due to tumor resection, trauma, congenital malformation, or alveolar bone resorption after teeth loss remain a significant challenge in oral reconstruction (1). Currently, autologous bone grafts have been considered the gold standard for the repair and reconstruction of maxillofacial bone defects, including critical-sized bone defects (2, 3). However, their clinical application is limited by disadvantages such as additional trauma at the donor site and limited bone availability (1, 4). Because of the limitations of autologous bone transplantation and other currently applied methods, bone tissue engineering that organically combines seed cells, bioactive factors, and biomaterials scaffolds is expected to provide an effective alternative approach for bone repair and reconstruction (5–7). Marrow mesenchymal stem cells (MSCs)-based therapies are considered viable alternatives with promising advantages for restoring the structure and function of damaged bone (5, 8). Bone marrow mesenchymal stem cells (BMSCs) are adult stem cells with high regeneration and multidirectional differentiation potential, which makes them ideal seed cells. Notably, research addressing the potential application of long non-coding RNAs (lncRNAs) in bone tissue engineering constructs for repair of bone defects is scarce. While the use of lncRNAs in combination with scaffolding has been reported in a few recent studies (8, 9), accumulating evidence shows that lncRNAs are involved in the osteogenic differentiation of various types of cells (9–12). Therefore, exploring the regulatory mechanisms related to lncRNAs’ roles in osteogenic differentiation could provide a theoretical basis for target discovery in applications within bone tissue engineering.

lncRNAs are a class of little or no protein-coding transcripts longer than 200 nucleotides (9). Improvements in sequencing technologies have led to the identification of thousands of IncRNAs in different cell types, including cells of cartilage and bone (13). Accumulating evidence has shown that lncRNAs are involved in the osteogenic differentiation of various types of cells, and several lncRNAs such as H19 (14, 15), MALAT1 (16, 17), MEG3 (18), and HOTAIR (19) have been found to regulate the osteogenesis of MSCs. lncRNAs are believed to regulate osteoblastic differentiation by mechanisms such as combining with RNA binding protein (RBP), interacting with sense transcripts, binding with EZH2, chromatin modification, binding to transcription factors, and acting as competitive endogenous RNA (ceRNA) (9, 11).

Super-enhancers (SEs) have been attracting attention since their concept was first proposed by Hnisz et al. in 2013 (20). SEs are a large cluster of enriched transcriptional activity enhancers that drive the expression of genes controlling cell identity. Compared with typical enhancers (TEs), SEs enrich a greater number of factors related to enhancer activity, such as Mediator1 (Med1), H3K27ac, H3K4me1, H3K4me2, and chromatin factors such as cohesin, p300, and CBP, RNA polymerase II (RNAPII), and therefore SEs display stronger transcriptional activation ability (21). Moreover, SEs can increase the transcription and production of enhancer-associated ncRNAs (eRNAs, elncRNAs) (21, 22).

SEs have been a particular focus of research in cell development and differentiation, and tumorigenesis. In recent years, accruing data has demonstrated a role of SEs in bone tissue regulation. Studies have reported that SEs are associated with various bone-related diseases, including osteosarcoma, Ewing sarcoma, chordoma, multiple myeloma, cartilage dysplasia, osteoporosis, rheumatoid arthritis, and osteoarthritis (23). These studies have typically identified disease-specific SEs and their target genes using bioinformatics, and then analyzed the functions of the target genes through experimental approaches (23). In conjunction, BRD4 and CDK7 based drugs targeting critical components of SEs have been developed to treat osteosarcoma, Ewing sarcoma, multiple myeloma, osteoarthritis, and other bone-related diseases (24–27). Specifically, Zhang et al. (24) have reported that the specific CDK7 inhibitor THZ2 could suppress the phosphorylation of RNAPII CTD and selectively suppress super-enhancer-related genes, leading to anti-osteosarcoma effects. Considering osteogenic differentiation, Yu et al. (28) have reported that the single-nucleotide polymorphisms (SNP)-unique ankylosing spondylitis (AS) SNP-adjacent SEs (SASEs)-mRNA network participated in the pathological osteogenesis of AS and enhanced the osteogenic differentiation ability of MSCs from AS patients (ASMSCs). Overall, research regarding the role of SEs in bone-related diseases and osteogenic differentiation has mainly focused on SEs-associated mRNAs but few studies have addressed SEs-associated lncRNAs. Thus, the function of SEs-associated lncRNAs (SE-lncRNAs) involved in osteogenic differentiation remains largely unknown.

SE-lncRNAs are typically transcribed from SE genomic regions or their adjacent regions which harbor specific chromatin states of activation senators, H3K4me1, H3K27ac, and related co-factors (such as P300, etc.) or interact with SEs (21, 29). Multiple functional studies have shown that elncRNAs are required for enhancer activity and target promoter transcriptional activity (30–33). In addition, Yan et al. (34) have applied genome-wide analysis and shown that in mouse embryonic stem cells (mESCs), lncRNA genes were preferentially located next to SEs, and consumption of SE-lncRNAs transcripts dysregulated the activity of nearby SEs. Many studies have highlighted the critical roles of SE-lncRNAs in tumorigenesis, cell development, and differentiation (22, 35–39). For example, LINC00162, an SE long non-coding RNA, was shown to bind to THRAP3 to inhibit the expression of PTTG1IP and promote the proliferation of bladder cancer cells (37). A super-enhancer-regulated lncRNA UCA1 in epithelial ovarian cancer (EOC) is shown to enhance the interaction between AMTO and YAP, activating YAP dephosphorylation and nuclear translocation, and promoting binding to TEAD to promote the expression of pro-oncogene signatures (38). CARMEN, an enhancer-associated lncRNA, was shown to cis-regulate the expression of miR-143/145 in adult cardiac precursor cells (CPCs) by producing CARMEN7, thereby regulating the differentiation of adult CPCs into smooth muscle cells (39). These findings highlight the basis for investigating the roles of SE-lncRNAs in the process of osteogenic differentiation.

In our previous studies, the SEs expressed before and after osteogenic differentiation D0 group (before osteogenic induction) and D14 group (day 14 after osteogenic induction) of human bone marrow mesenchymal stem cells (hBMSCs) were identified by chromatin immunoprecipitation sequencing (ChIP-seq) of H3K27ac (40). The associated genes of the specific SEs in the D14 group were analyzed using bioinformatics and quantitative real-time polymerase chain reaction (qRT-PCR), and the SE-lncRNA LINC01485 was found to show significant differences in expression levels before and after osteogenic differentiation. Therefore, the role and regulatory mechanisms of LINC01485 involvement in the differentiation of hBMSCs were considered to merit further research and are addressed in the present investigation.

## Materials and Methods

### Annotation and Prediction of SE-Associated Genes

SEs were assigned to the expressed transcripts, and the transcription start site (TSS) closest to the center of the enhancer was used to identify neighboring genes (20, 24, 41). AnnotatePeaks.pl, a Hypergeometric Optimization of Motif EnRichment (Homer, version 4.11) application for peak annotation, was used to link peaks to the neighboring genes (42, 43).

### Culture and Osteogenic Differentiation of hBMSCs

hBMSCs were purchased from Procell Life Science & Technology (CP-H166, Wuhan, China) and Cyagen Biosciences (HUXMA-01001, Guangzhou, China) and cultured in MSC medium (Cyagen, China) supplemented with 10% fetal bovine serum (FBS), 1% penicillin, and streptomycin, 1% glutamine (Cyagen, China) in humidified air of 5% CO_2_ at 37°C. The purchased cells were accompanied by quality reports, including flow cytometry identification, which revealed that the hBMSCs were positive for CD29, CD44, CD73, and CD105, and negative for CD34, CD11b, and CD45. The purchased hBMSCs could differentiate into osteoblasts, adipocytes, and chondrocytes under specific inductive conditions. When cells reached 80-90% confluence, subculture was performed at a ratio of 1:2 or 1:3, and the medium was replaced every 2 days. After being cultured to P2-P4, the cells were plated in a 6-well plate at a density of about 1×10^5^ cells/well. When cell confluence reached roughly 70%, hBMSCs osteogenic induction medium (Cyagen, China) containing dexamethasone, vitamin C, and β-sodium glycerophosphate was added, and was changed every 3 days.

### qRT-PCR

Total RNA was extracted from the cells using TRIzol reagent (Accurate Biotech, Hunan, China). RNA purity and concentration were assessed by NanoDrop 2000 instrument (Thermofisher, US). For the qRT-PCR quantification of mRNAs and lncRNAs, 1000 ng RNA was reverse transcribed into cDNA using the Evo M-MLV RT kit with gDNA Clean for qPCR (Accurate Biotech, China). Reverse transcription of miRNAs was performed using Bulge-Loop SCRIPT Reverse Transcription Kit (RiboBio, Guangzhou, China). qRT-PCR was performed using SYBR Green Premix PCR kit (Accurate Biotech, China) on a CFX Connection Real-Time System (Bio-Rad, California). According to the manufacturer’s protocol, the qRT-PCR reaction program for mRNAs and lncRNAs was set as follows: initial activation at 95°C for 30 s, followed by 40 cycles at 95°C for 5 s and 63°C for 30 s, while the cycling conditions for miRNAs were as follows: initial activation at 95°C for 10 min, followed by 40 cycles at 95°C for 2 s, 60°C for 20 s, and 70°C for 10 s. GAPDH was used as an endogenous reference for lncRNAs and mRNAs, while U6 was used to normalize the expression of miRNAs. The 2^−ΔΔCT^ method was used to calculate the relative expression level of each gene. All reactions were performed in duplicate to ensure reliability and validity. All primers were obtained from Tsingke (Beijing, China) or RiboBio (Guangzhou, China). The sequences of the primers used are listed in **Supplementary Tables S2**, **S3**.

### Western Blot

Total protein was extracted by RIPA lysate (Cwbio, Jiangsu, China) containing protein inhibitors and phosphatase inhibitors (Cwbio, China) and was quantified by bicinchoninic acid (BCA) protein assay kit (Cwbio, China). A total of 20 µg protein from each sample was separated by 10% SDS-PAGE gel at 80V (stacking gel)/120V (resolving gel) for about 2 h and transferred onto polyvinylidene fluoride (PVDF) membranes (Millipore, US) with a diameter of 0.45 um at 250 mA for 150 min. The membrane was blocked with 5% non-fat milk at room temperature for 1 h, washed with TBST (0.1% Tween-20 in Tris-buffered saline (TBS)) and incubated overnight in the primary antibody at 4°C. The primary antibodies were as follows: RUNX2 (1:1000, Cell Signaling Technology, Cat# 12556s), Osterix (1:1000, BOSTER, Cat# A02077-1), COL1A1 (1:1000, BOSTER, Cat# BA0325), OPN (1:1000, Abcam, Cat# ab8448), and GAPDH (1:20000, Proteintech, Cat# 60004–1-Ig). The membrane was washed with TBST three times for 10 min each and then incubated with an HRP-conjugated secondary antibody (Goat Anti-Mouse IgG, 1:5000, Proteintech, Cat # SA00001-1; Goat Anti-Rabbit IgG, 1:5000, Proteintech, Cat# SA00001-2) at room temperature for 1 h followed by washing with TBST three times for 10 min each. Immune complexes were detected using an ECL kit (Merck Millipore, Germany) with a chemiluminescence imaging system (Bio-Rad, US). The density data of each specific protein was normalized to that of GAPDH and analyzed using Image J software (Media Cybernetics, US).

### Alkaline Phosphatase (ALP) Staining and ALP Activity Detection

hBMSCs plated in 6-well plates were subjected to osteogenic induction for 7 days, then washed twice with phosphate-buffered saline (PBS), and fixed with 4% paraformaldehyde (PFA) for 15 min. After washing with PBS, the cells were stained with ALP staining solution (Beyotime, Shanghai, China) according to the manufacturer’s instructions. After 24 h of staining, the cells were photographed under a microscope (Leica, DMIRB, Germany). In addition, the cells were added with lysates (Beyotime, China), which were collected and used to detect ALP activity using an ALP activity test kit (Beyotime, China). The absorbance at 450 nm was examined.

### Alizarin Red S (ARS) Staining and ARS Quantification Assay

hBMSCs were subjected to osteogenic induction for 14 days, washed twice with PBS, and fixed with 4% PFA for 15 min. Next, they were washed with diH_2_O and stained with 40 mM ARS (ScienCell, US) at 37°C for 15 min. After dyeing, the cells were washed with diH_2_O, and images were obtained under the microscope (Leica, DMIRB, Germany). The stained cells were added with 10% acetic acid and 10% ammonium hydroxide from an Alizarin Red S staining quantification assay kit (ScienCell, US). The absorbance at 405 nm was determined and used to analyze the ARS concentration.

### Lentivirus Construction and Cell Transduction

The full-length sequences of LINC01485, LINC01485 short hairpin (sh) RNA targeting LINC01485 (sh-LINC01485), and scrambled control shRNA (sh-NC) were inserted into the GV vector, and the three target plasmid vectors and an empty plasmid vector were transfected into 293T cells with plasmids Helper 1.0 and Helper 2.0, respectively. The cell supernatant was collected to obtain the virus, and the virus was concentrated, purified, and detected. The inserted sequence was confirmed by sequencing analysis. This work was done by GeneChem (GeneChem, Shanghai, China). hBMSCs were plated in a 6-well plate at a density of 6×10^4^ cells/well. When the cells became adherent to the wall about 24 h later, and the confluence reached approximately 50%, the cells were infected with lentiviruses at an MOI of 40 for 2-3 days. Then, 3 μg/mL puromycin (Solarbio, Beijing, China) was utilized for screening for 2 days. Finally, the cells were collected to test the overexpression and interference efficiency of LINC01485 and used for further experiments. The sequence of LINC01485 RNAi is listed in **Supplementary Table S4**.

### Bioinformatics Analysis of Targeting Relationship Between lncRNA-miRNA and miRNA-mRNA

lncRNA-bound miRNAs and miRNA-targeted mRNAs were predicted using TargetScan and Miranda databases, and the predicted results of the two databases were intersected. First, the target mRNAs of miRNA associated with osteogenesis were selected from the relationship pairs with the highest binding score. Then the RNA antisense purification (RAP) assay was performed to determine the miRNA that was finally interacting with LINC01485.

### MiRNA Transfection

hBMSCs were seeded in the 6-well plate at a density of 6×10^4^, and the cells reached 70% confluence after about 48 h. According to the manufacturer’s instructions, miR-619-5p mimic and miRNA mimic NC (RiboBio, Guangzhou, China) were transfected with Lipofectamine 3000 transfection reagent (Invitrogen, US) at a concentration of 50 nM, and miR-619-5p inhibitor, miRNA inhibitor NC (RiboBio, Guangzhou, China) at a concentration of 100 nM. RNA was extracted 24 h after transfection, the protein was extracted 48 h for detection, and osteogenic induction solution was added for osteogenic induction differentiation. Transfection was performed again after 3 days. MiR-619-5p mimic and miR-619-5p inhibitor sequence are listed in **Supplementary Table S5**.

### FISH Assay

Before and after osteogenic induction, hBMSCs in 6-well plates were fixed with 4% PFA for 15 min upon reaching a cell density of 70-80%. The cells were washed with DEPC water twice for 5 min each time, then added protease K and incubated at 55°C for 5 min for digestion. At room temperature, the cells were fixed again with 1% PFA for 10 min and washed three times with pre-cooled alcohol at -20°C. Pre-hybridization was performed with a 100 μL pre-hybridization solution dropped onto slices at 37°C for 30 min. The prepared LINC01485 probe (Axl-bio, Guangzhou, China) was denatured at 73°C for 8 min. The probe and hybrid solution mixture was added to the sections and hybridized overnight at 42°C. After washing with hybridization solution and PBS, according to the instructions of the FISH test kit (Axl-bio, Guangzhou, China), 4,6-diamidino-2-phenylindole (DAPI) was added for staining, in light avoid conditions for 10 min, followed by washing with PBS 3 times, 5 min each. Then, anti-fluorescence attenuated tablets were used to seal the tablets, and the images were obtained with laser scanning confocal microscopy (Carl Zeiss AG, Germany). LINC01485 FISH probe is listed in **Supplementary Table S6**.

### RAP Assay

For this experiment, the RAP kit (Axl-bio, Guangzhou, China) was employed.

RAP uses biotinylated probes that bind to target RNAs and miRNAs, which may subsequently be extracted, reverse transcribed to cDNA, and detected by qRT-PCR. A total of 10^7^ hBMSCs were washed in PBS and UV irradiated at 254 nm (0.15 J cm^-2^) for cross-linkage, 1 mL lysis buffer was used to lyse the cells, and a 0.4-mm syringe was used to homogenize them completely. The lncRNA-RAP system received two separate 25-bp biotinylated antisense probes (0.2 nmol), as well as one 26-bp biotinylated antisense probe (0.2 nmol) targeting the adapter sequence. The probes were denatured at 65°C for 10 min and hybridized at room temperature for 2 h. There were 200μL streptavidin-coated magnetic beads added, washing was employed to remove non-specifically bound RNAs, and Trizol reagent was used to extract miRNAs directly interacting with LINC01485. The miRNAs were reverse transcribed and binding strength was determined using qRT-PCR. The LINC01485 RAP probe sequence is listed in **Supplementary Table S7**.

### Luciferase Assay

HEK293T cells were purchased from ATCC cell bank, cultured, and amplified with DMEM (Gibco, US) complete medium containing 10% FBS (Gibco, US). HEK293T cells were plated in 6-well plates at a density of 2×10^6^ cells/well. After 16 h, the cell confluence reached about 80%. The possible binding sites of LINC01485 and miR-619-6p were predicted using bioinformatics tools, and the predicted LINC01485 binding sequence and mutated sequence were constructed into psicheck2 reporter plasmid. At the same time, the full-length sequence of LINC01485 was inserted into the pcDNA3.1 plasmid. The plasmids and miR-619-5p mimic or miRNA NC were co-transfected into HEK293T cells with Lipofectmin 3000 transfection reagent (Invitrogen, US). The medium was changed 6 h after transfection, and the culture was continued until 48 h after adding the complete medium. The medium was removed, washed twice with PBS, and 1×PLB lysate (Promega, US) was added for incubation at room temperature for 15 min. A total of 20 μL lysate, 100 µL LARII, and 100 µL stop Glo buffer (Promega, US) were added to a 96-well plate, and luciferase activity was detected using a microplate reader (BioTek, US).

### Statistical Analysis

All experiments were carried out three times independently. Data were presented as mean ± standard deviation (SD) based on three replicates. Unpaired two-tailed Student’s t-test was used to examine differences between groups. All data were statistically analyzed using GraphPad Prism 8.0 (GraphPad Software, US). Statistical significance was defined as a value of *p*<0.05 (two-sided).

## Results

### LncRNAs in Specific SE-Associated Genes in the D14 Group

Using HOMER software analysis, the genes closest to the SEs were selected as the SE-associated genes. **Supplementary Table S1** shows lncRNAs in the D14 group specific SE-associated genes. qRT-PCR results showed that the expression levels of 4 lncRNAs were significantly increased after osteogenic differentiation (**Supplementary Figure S1**).

### LNC01485 Expression Was Up-Regulated During Osteogenic Differentiation of hBMSCs

**Figure 1:**
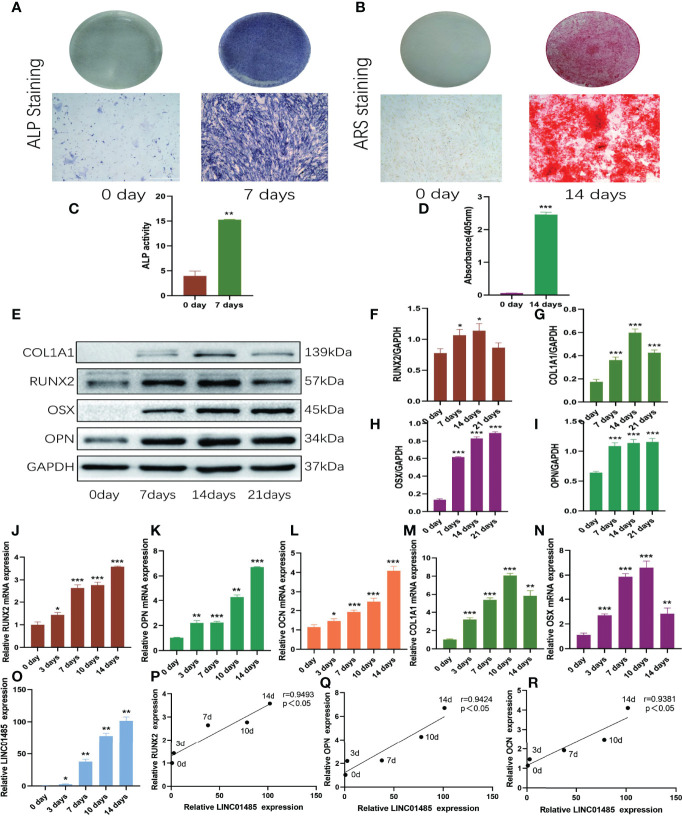
LNC01485 expression was up-regulated during osteogenic differentiation of hBMSCs. **(A, C)** ALP staining **(A)** and ALP activity assay **(C)** of hBMSCs before osteogenic induction and 7 days after induction. **(B, D)** hBMSCs were stained with Alizarin Red S **(B)** before osteogenic induction and at 14 days after induction, and the staining results were analyzed semi-quantitatively **(D)**. **(E-I)** The protein expression levels of RUNX2 **(E, F)**, COL1A1 **(E, G)**, OSX **(E, H)**, and OPN **(E, I)** level on Days 0, 7, 14, and 21 of osteogenic induction were detected by Western blot and quantified analysis by normalized to GAPDH. **(J-O)** The mRNA expression levels of RUNX2 **(J)**, OPN **(K)**, OCN **(L)**, COL1A1 **(M)**, OSX **(N)**, and LINC01485 **(O)** before and after osteogenic differentiation were determined by qRT-PCR. **(P-R)** Expression correlation analysis between LINC01485 and osteogenic genes RUNX2 **(P)**, OPN **(Q)**, and OCN **(R)** during osteogenic differentiation. *p < 0.05, **p < 0.01, ***p < 0.001.

At first, the osteogenic differentiation of hBMSCs was confirmed by ALP activity assay and ALP and ARS staining. ALP staining (**Figure 1A**) on Day 7 after induction and ARS staining (**Figure 1B**) on Day 21 after induction showed positive staining results. The ALP activity was significantly enhanced after osteogenic induction on the seventh day (**Figure 1C**). Alizarin Red semi-quantitative analysis showed that the content of Alizarin Red bound to calcium nodules increased significantly 14 days after osteogenesis induction (**Figure 1D**). Western blot and qRT-PCR assays were used to determine the expression levels of runt-related transcription factor (RUNX2), collagen type I alpha 1 chain (COL1A1), osterix (OSX), osteocalcin (OCN), and osteopontin (OPN) during the process of osteogenic induction of hBMSCs. Western blot results showed that protein levels of RUNX2 (**Figures 1E, F**), COL1A1 (**Figures 1E, G**), OSX (**Figures 1E, H**), and OPN (**Figures 1E, I**) were up-regulated after osteogenic induction as compared to uninduced cells. The qRT-PCR results revealed that RUNX2 (**Figure 1J**), OPN (**Figure 1K**), and OCN (**Figure 1L**) increased gradually during 14 days of osteogenic induction, and COL1A1 (**Figure 1M**) and OSX (**Figure 1N**) reached their peak on the tenth day. Compared with the uninduced cells, the expression levels of these osteogenic factors were significantly up-regulated (*p*<0.05). These results revealed that the osteogenic induction of hBMSCs *in vitro* was achieved successfully.

The mRNA expression levels of LINC01485 were determined by qRT-PCR during the osteogenic differentiation of hBMSCs. The expression of LINC01485 gradually increased over time during 14 days of osteogenic induction (**Figure 1O**). The expression trend of LINC01485 was consistent with that of osteogenic genes RUNX2, OPN, and OCN; therefore, the correlation between LINC01485 and osteogenic-related genes was analyzed using Spearman’s correlation analysis. LINC01485 was evident as significantly positively correlated with RUNX2 (**Figure 1P**), OPN (**Figure 1Q**), and OCN (**Figure 1R**), suggesting that LINC01485 might be involved in osteogenesis regulation.

### LINC01485 Regulates Osteogenic Differentiation of hBMSCs

**Figure 2:**
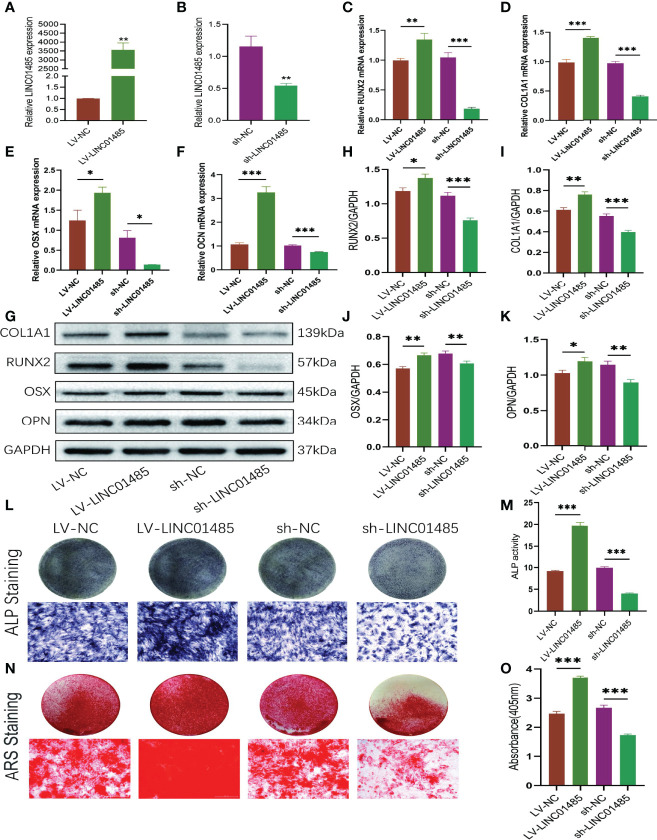
LINC01485 regulates osteogenic differentiation of hBMSCs. **(A, B)** The overexpression **(A)** and interference **(B)** efficiency of LINC01485 was determined by qRT-PCR in hBMSCs after transduction with LV-LINC01485 and sh-LINC01485. **(C–F)** The mRNA levels of RUNX2 **(C)**, COL1A1 **(D)**, OSX **(E)**, and OCN **(F)** after 14 days of osteogenic induction in hBMSC infected with lentivirus by qRT-PCR. **(G, H)** Western blot analysis of the RUNX2 **(G, I)**, COL1A1 **(G, I)**, OSX **(G, J)**, and OPN **(G, K)** protein expression in hBMSCs infected with lentivirus after osteogenic induction 14 days later and the corresponding gray value quantitative analysis. **(L, M)** ALP staining **(L)** and ALP activity **(M)** of hBMSC cells infected with lentivirus after 7 days of osteogenic induction. **(N, O)** Alizarin Red S staining **(N)** and semi-quantitative analysis **(O)** of infected hBMSCs with lentivirus after 14 days osteogenic induction. *p < 0.05, **p < 0.01, ***p < 0.001.

To explore the effects of LINC01485 on osteogenic differentiation of hBMSCs *in vitro*, we infected cells with LINC01485 overexpression and LINC01485 knockdown lentivirus to maintain continuous expression levels of LINC01485. qRT-PCR was performed to examine the overexpression and interference efficiency of LINC01485. The results verified that the level of LINC01485 expression in the LINC01485-overexpression group was significantly higher than that in the negative control group after transduction (**Figure 2A**). In contrast, LINC01485 was down-regulated after transduction of sh-LINC01485, as compared with the scrambled group (**Figure 2B**). Subsequently, qRT-PCR and western blot assays were used to determine the expression levels of osteogenic specific factors in the infected groups at mRNA and protein levels after 14 days of osteogenic induction. As shown in **Figures 2C–K**, LINC01485 overexpression markedly enhanced the expression of RUNX2 (**Figure 2C**), COL1A1 (**Figure 2D**), OSX (**Figure 2E**), and OCN (**Figure 2F**) at the mRNA level and promoted the expression of RUNX2 (**Figures 2G, H**), COL1A1 (**Figures 2G, I**), OSX (**Figures 2G, J**), and OPN (**Figures 2G, K**) at the protein levels. In contrast, LINC01485 knockdown produced the opposite effects (**Figures 2C–K**). Furthermore, ALP activity detection and ALP staining revealed that the up-regulation of LINC01485 enhanced ALP staining (**Figure 2L**) and the ALP activity (**Figure 2M**), while the down-regulation of LINC01485 reduced ALP staining (**Figure 2L**) and inhibited ALP activity (**Figure 2M**). ARS staining with LINC014845 overexpression led to increase in the mineralized bone matrix as compared to the control group (**Figures 2N, O**), whereas sh-LINC01485 decreased calcium nodules (**Figures 2N, O**). These results manifested that LINC01485 could promote osteogenic differentiation of hBMSCs *in vitro*.

### LINC01485 Acts As A Sponge of miR-619-5p in the Osteogenesis of hBMSCs

**Figure 3:**
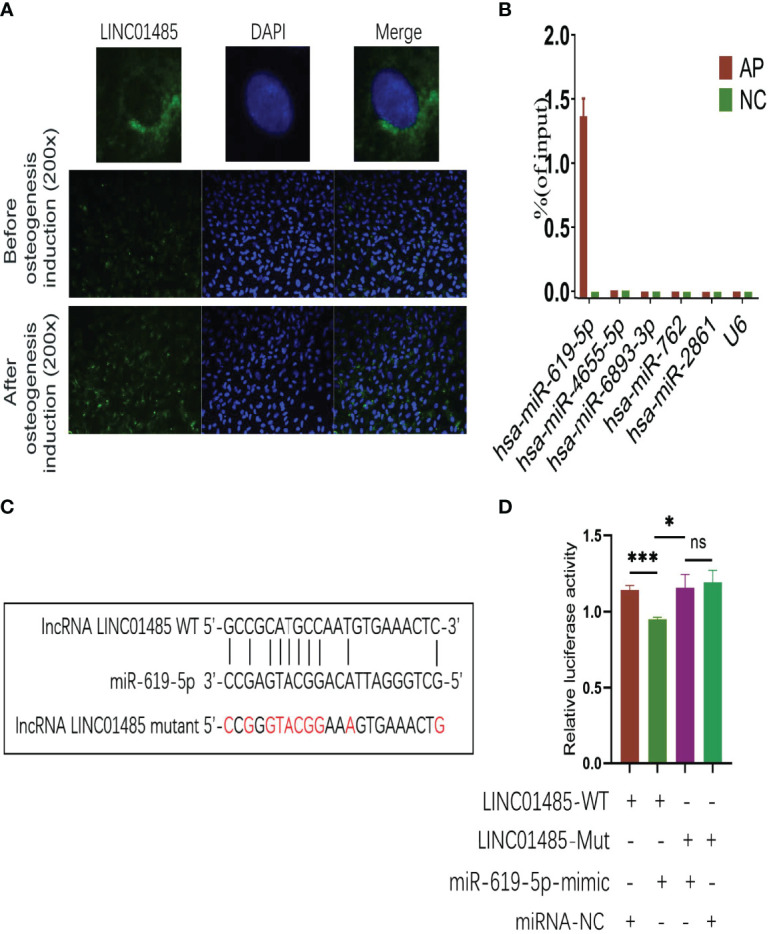
LINC01485 acts as a sponge of miR-619-5p in the osteogenesis of hBMSCs. **(A)** FISH assay for LINC01485 before and after osteogenic induction in hBMSCs. **(B)** Percentage of purified miRNAs identified in the RAP assay relative to the input group detected by qRT-PCR. **(C)** Binding site prediction of LINC01485 and miR-619-5p. **(D)** Luciferase activity of LINC01485-WT and LINC01485-Mut upon transfection of miRNA NC or miR-619-5p mimic into HEK293T cells. ns, no significance. *p < 0.05, ***p < 0.001.

To investigate the molecular mechanisms of LINC01485 regulation of osteogenic differentiation of hBMSCs, we first evaluated the cellular localization of LINC01485. FISH assay results determined that LINC01485 was mainly expressed in the cytoplasm (**Figure 3A**). This finding is consistent with the results reported by Zhou et al. (44) showing LINC01485 as primarily located in the cytoplasm of gastric cancer cells. In addition, FISH results also suggested greater LINC01485 fluorescence in hBMSCs after osteogenic induction as compared with uninduced cells (**Figure 3A**).

Recent research (12) has shown that lncRNA located in the cytoplasm plays a role in the osteogenic differentiation of MSCs through cross-talk with miRNA. Thus, we used Miranda and TargetScan databases to predict miRNAs that might bind to LINC01485. A Venn diagram depicted the intersection. To further screen target miRNAs, an RAP assay of LINC01485 was conducted to determine the expression levels of several miRNAs that displayed high predicted scores. RAP results showed that among 5 miRNAs with high scores, miR-619-5p was evident in the AP group as compared with the Input group without RAP probe, while the other miRNAs showed negative results in the AP group (**Figure 3B**). The electrophoretic patterns of qRT-PCR products in the RAP experiment also confirmed this result (**Supplementary Figure S2**). Thus, the RAP results indicated that LINC01485 efficiently pulled down miR-619-5p but did not bind to several other miRNAs.

Furthermore, the binding sites of LINC01485 and miR-619-5p were predicted using bioinformatics (**Figure 3C**). Thereafter, we constructed LINC01485 wild-type (WT) and LINC01485 mutant (MUT) luciferase receptor plasmids based on the putative binding sites. The result of luciferase activity analysis indicated that the luciferase activity of LINC01485-WT reporter vector was significantly inhibited by miR-619-5p mimic, while luciferase activity of LINC01485-MUT type was not affected by miR-619-5p mimic (**Figure 3D**).

### MiR-619-5p is Down-Regulated and Inhibits Osteogenesis During Osteogenic Induction of hBMSCs

**Figure 4:**
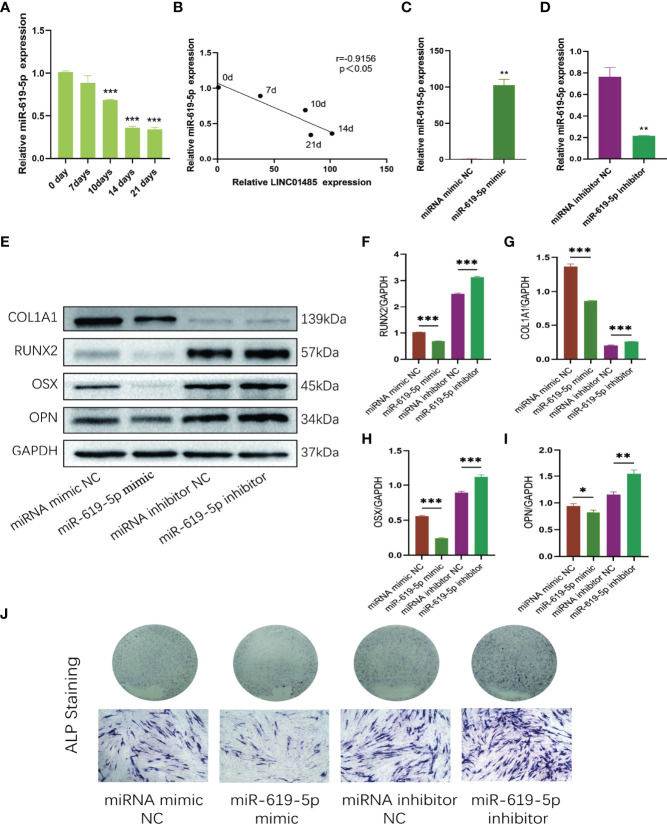
MiR-619-5p is down-regulated and inhibits osteogenesis during osteogenic induction of hBMSCs. **(A)** The relative expression levels of miR-619-5p before and after osteogenic differentiation were determined by qRT-PCR. **(B)** Correlation analysis of LINC01485 and miR-619-5p expression levels during osteogenic differentiation. **(C, D)** The mRNA level of miR-619-5p in hBMSCs transfected with miR-199a-5p mimic **(C)** and miR-199a-5p inhibitor **(D)** by qRT-PCR. **(E–I)** Western blot analysis of the RUNX2 **(E, F)**, COL1A1 **(E, G)**, OSX **(E, H)**, and OPN **(E, I)** protein expression in hBMSCs transfected with miR-619-5p mimic, miRNA mimic NC, miR-619-5p inhibitor, and miRNA inhibitor NC after osteogenic induction and the corresponding gray value quantitative analysis. **(J)** ALP staining analysis of hBMSCs transfected with miR-619-5p mimic, miRNA mimic NC, miR-619-5p inhibitor, and miRNA inhibitor NC after osteogenic induction. *p < 0.05, **p < 0.01, ***p < 0.001.

Since LINC01485 could sponge miR-619-5p to promote osteogenic differentiation, we next studied the expression of miR-619-5p in the osteogenic process and its effect on osteogenic differentiation. qRT-PCR analysis showed that the mRNA level of miR-619-5p decreased gradually during osteogenic differentiation (**Figure 4A**), which was negatively correlated with the expression trend of LINC01485 (**Figure 4B**). Subsequently, miR-619-5p mimic, miRNA mimic negative control (miRNA mimic NC), miR-619-5p inhibitor, and miRNA inhibitor negative control (miRNA inhibitor NC) were synthesized and transfected into hBMSCs for osteogenic induction, followed by western blot and ALP staining assay. qRT-PCR results showed that the expression level of miR-619-5p in cells transfected with miR-619-5p mimic increased to about 100-fold higher than that in mimic NC (**Figure 4C**). In contrast, the expression level of miR-619-5p in cells transfected with miR-619-5p inhibitor was about 72% lower than that in inhibitor NC (**Figure 4D**). MiR-619-5p mimic significantly reduced the protein levels of RUNX2 (**Figures 4E, F**), COL1A1 (**Figures 4E, G**), OSX (**Figures 4E, H**), and OPN (**Figures 4E, I**), and weakened ALP staining (**Figure 4J**). At the same time, inhibition of miR-619-5p led to an increase in RUNX2 (**Figures 4E, F**), COL1A1 (**Figures 4E, G**), OSX (**Figures 4E, H**), and OPN (**Figures 4E–I**) protein expression and enhanced ALP staining (**Figure 4J**). These results indicated that miR-619-5p overexpression could inhibit osteogenic differentiation, and inhibition of miR-619-5p could promote the osteogenesis of hBMSC. This is contrary to the effect of LINC01485 on osteogenic differentiation and adds to the evidence that LINC01485 promotes osteogenic differentiation through the LINC01485/miR-619-5p axis.

### RUNX2 Is A Direct Target of miR-619-5p

**Figure 5:**
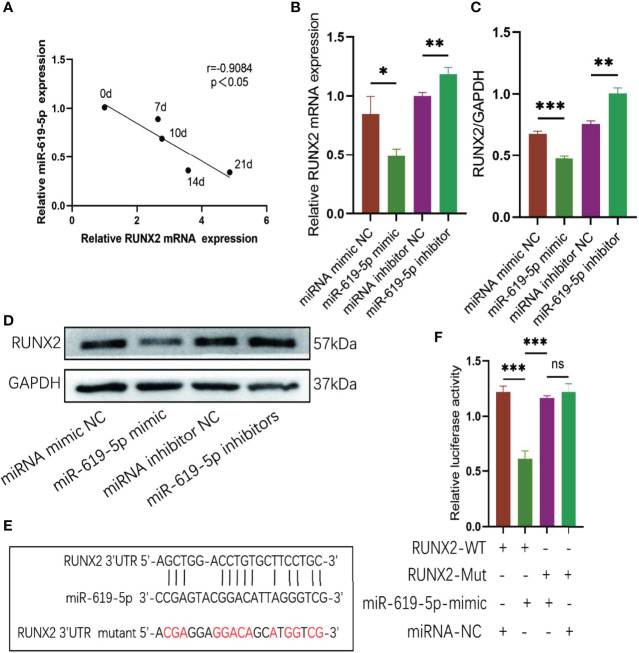
RUNX2 is a direct target of miR-619-5p. **(A)** Correlation analysis of RUNX2 and miR-619-5p expression levels during osteogenic differentiation. **(B)** The mRNA expression of RUNX2 in hBMSCs transfected with miR-619-5p mimic or miR-619-5p inhibitor was determined using qRT-PCR. **(C, D)** The RUNX2 protein level in hBMSCs transfected with miR-619-5p mimic or miR-619-5p inhibitor was determined using Western blotting **(C)** and the gray value quantitative analysis **(D)**. **(E)** The putative binding sites of miR-619-5p in wild type and mutant RUNX2 3′-UTR. **(F)** Luciferase activity of RUNX2-WT and RUNX2-Mut upon transfection of miRNA NC or miR-619-5p mimic into HEK293T cells. ns, no significance. *p < 0.05, **p < 0.01, ***p < 0.001.

We predicted the target genes of miR-619-5p using Miranda and TargetScan databases and found that miR-619-5p could bind to the osteogenic gene RUNX2. The mRNA level of RUNX2 increased gradually during osteogenic differentiation, which was negatively correlated with the expression of miR-619-5p (**Figure 5A**). In addition, Western blot and qRT-PCR analysis showed that protein and mRNA levels of RUNX2 decreased when hBMSCs were transfected with miR-619-5p mimic, while protein and mRNA levels of RUNX2 increased by the treatment with the miR-619-5p inhibitor (**Figures 5B–D**). Moreover, the results of bioinformatic predictions showed that miR-619-5p had a binding site at the 3’UTR of RUNX2, and the binding sequence is shown in **Figure 5E**. The dual-luciferase assay showed that luciferase activity of RUNX2-WT was significantly down-regulated by miR-619-5p mimic, but the luciferase activity of RUNX2-MUT was not affected by miR-619-5p (**Figure 5F**). These results confirmed that miR-619-5p targets RUNX2, inhibiting RUNX2 expression by binding to the 3’UTR of RUNX2.

### LINC01485 Acts As A ceRNA of miR-619-5p to Regulate RUNX2 and Osteogenic Differentiation

**Figure 6:**
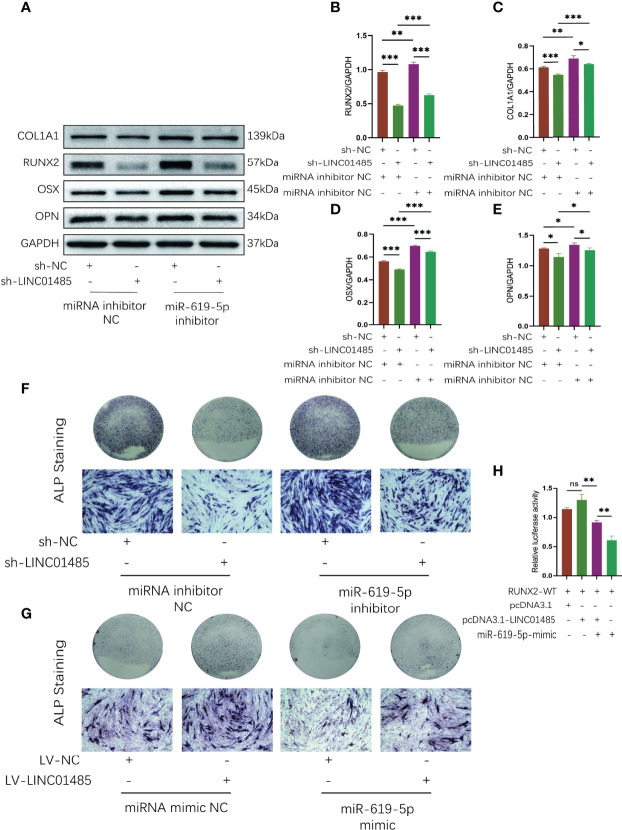
LINC01485 acts as a ceRNA of miR-619-5p to regulate RUNX2 and osteogenic differentiation. **(A–E)** Western blot analysis of the RUNX2 **(A, B)**, COL1A1 **(A, C)**, OSX **(A, D)**, and OPN **(A, E)** protein expression in hBMSCs infected with sh-NC or sh-LINC01485 lentivirus along with miRNA inhibitor NC or miR-619-5p inhibitor after osteogenic induction and the corresponding gray value quantitative analysis. **(F)** ALP staining analysis of hBMSCs infected with sh-NC or sh-LINC01485 lentivirus along with miRNA inhibitor NC or miR-619-5p inhibitor after osteogenic induction. **(G)** ALP staining analysis of hBMSCs infected with LV-NC or LV-LINC01485 lentivirus along with miRNA mimic NC or miR-619-5p mimic after osteogenic induction. **(H)** Luciferase activity of RUNX2-WT upon transfection of pcDNA3.1, pcDNA3.1-LINC01485, or miR-619-5p mimic into HEK293T cells. ns, none significance. *p < 0.05, **p < 0.01, ***p < 0.001.

A series of rescue assays was performed to verify whether there is a regulatory relationship among LINC01485, miR-619-5p, and RUNX2. First, we transfected miR-619-5p inhibitor or miRNA inhibitor NC into sh-LINC01485 or sh-NC stable hBMSCs constructed in advance. Western blot results showed that after osteogenic induction of hBMSCs in the LINC01485 interference group, the protein levels of RUNX2 (**Figures 6A, B**), COL1A1 (**Figures 6A, C**), OSX (**Figures 6A, D**), and OPN (**Figures 6A, E**) were decreased, and ALP staining was reduced as compared with the control group (**Figure 6F**). However, miR-619-5p inhibitor treatment compensated for the effect of LINC01485 knockdown on osteogenic differentiation, and the protein levels of RUNX2 (**Figures 6A, B**), COL1A1 (**Figures 6A, C**), OSX (**Figures 6A, D**), and OPN (**Figures 6A, E**) were elevated, and ALP staining was more pronounced (**Figure 6F**). Then, miR-619-5p mimic or miRNA mimic NC was transfected into LV-LINC01485 or LV-NC stable hBMSCs for osteogenic differentiation induction. miR-619-5p mimic reduced ALP staining as compared with miRNA mimic NC, but the ALP staining increased after co-transfection of LV-LINC01485 (**Figure 6G**). Luciferase assays indicated that miR-619-5p mimic reduced the luciferase activity of RUNX2-WT, while the co-transfection of LINC01485 overexpressed plasmid with miR-619-5p mimic restoring the luciferase activity of RUNX2-WT (**Figure 6H**). RUNX2-WT luciferase activity was higher in the LINC01485 overexpressed plasmid transfection group than in RUNX2-WT transfected with the empty plasmid but decreased when miR-619-5p mimic was added (**Figure 6H**). Overall, these *in vitro* results suggest that LINC01485, miR-619-5p, and RUNX2 constitute a ceRNA network, and LINC01485 competes with miR-619-5p to regulate RUNX2 expression, thereby regulating the bone formation of hBMSCs.

## Discussion

Maxillofacial critical size bone defects caused by several etiological factors pose challenges in successful reconstruction. Therefore, exploring the regulatory mechanisms underlying BMSCs osteogenic differentiation can provide a theoretical basis and target discovery for bone tissue engineering. SEs comprise a genomic region composed of activity enhancer clusters enriched with many factors related to enhancer activity. Compared with TE related genes and genomic regions, SEs are found in all cell lines spanning a highly cell type-specific genomic domain and are associated with master cell-type-specific regulatory genes (45–47). Recent studies have shown that SE-associated genes are involved in multiple bone-related diseases. For instance, *TBXT* has been found to play an essential role in chordoma pathogenesis (48), and was associated with SE, where transcriptional cyclin-dependent kinase(CDK)inhibitors could downregulate the expression of brachyury/TBXT. Similarly, Lin et al. (49) reported that MEIS1, an SE-associated oncogene, promotes the malignant development of Ewing sarcoma by synergistic activation of APCDD1 transcription with EWS-FLI1. These studies indicate that SE-associated mRNAs can play essential roles in bone regulation. In this study, we identified the SE-lncRNA LINC01485 through bioinformatics investigation and found that it was elevated during the osteogenic differentiation of hBMSCs.

SE-lncRNAs are transcribed from or interact with SEs. A higher percentage of SEs are reported to generate eRNAs termed seRNAs compared to TEs (50). LINC01485 is located on chromosome 5q35.2 and has been shown to be an oncogenic gene. In earlier work, LINC01485 has been reported to promote the growth and migration of gastric cancer cells by inhibiting EGFR ubiquitination and activating its downstream Akt signal (44). Additionally, LINC01485 was reported among the 6 lncRNAs that emerged as prognostic indicators for colorectal cancer (51). However, no studies have explored the role of LINC01485 in osteogenic differentiation. The present study demonstrated that the overexpression of LINC01485 could promote osteogenic differentiation as indicated by increase in ALP activity and ALP staining, along with enhanced production of calcified nodules and expression of osteoblast differentiation markers, while the knockdown of LINC01485 exerted the opposite effects on osteogenic differentiation. These results indicated that LINC01485 could positively regulate the osteogenic differentiation of hBMSCs.

elncRNAs perform regulatory transcriptional functions by -cis and -trans mechanisms (21). The roles of seRNAs include chromatin loops stabilization, and transcription factor recruitment among others, and thus seRNAs mediate various cellular activities in the cytoplasm (52). To further explore the molecular mechanism of LINC01485 involvement in osteogenic differentiation process, we first identified the subcellular localization of LINC01485 and found that LINC01485 is mainly localized in the cytoplasm, consistent with the results reported by Zhou et al. (44). The cellular localization of lncRNAs is closely related to its mechanism of action. Cytoplasmic lncRNAs can regulate the stability and transcription of mRNA and exert their functions mainly through transcription or translation regulation (53). For instance, in related studies (38, 54) super-enhancer-regulated lncRNA UCA1 has been found to serve as an endogenous competitive RNA binding miR-193a-3p to regulate ERBB4, thereby promoting proliferation and colony formation of lung cancer cells. In yet another finding, Wang et al. (55) reported that UCA1 could repress the host immune system, stimulate the proliferation and migration of gastric cancer cells, and inhibit apoptosis of gastric cancer cells by directly interacting with miR-26a/b, miR-193a, and miR-214 anti-tumor miRNAs to up-regulate PDL1 expression. Here, we predicted the binding of LINC01485 to miR-619-5p using in-silico approaches and verified this relationship by performing RAP and luciferase assays. Cross-talk between lncRNA and miRNA has been found to be involved in a variety of osteogenic signaling pathways, including TGF/BMP-SMAD dependent and non-dependent, and the Wnt/β-catenin pathways (56–58). Specifically, miR-619-5p has been shown to improve pancreatic cancer sensitivity to gemcitabine by targeting Pygo2 and activating the Wnt/β-catenin pathway (59). The Wnt/β-catenin pathway is shown to play a critical role in regulating the osteogenic differentiation of hMSCs (60, 61). Therefore, here we hypothesized that miR-619-5p might be involved in osteodifferentiation of hBMSCs by activating the osteogenesis-related Wnt/β-catenin pathway and subsequently demonstrated that miR-619-5p, as a negative regulator of osteogenic differentiation, could inhibit the protein expression levels of RUNX2, COL1A1, OSX, and OPN, and reduce ALP staining.

**Figure 7:**
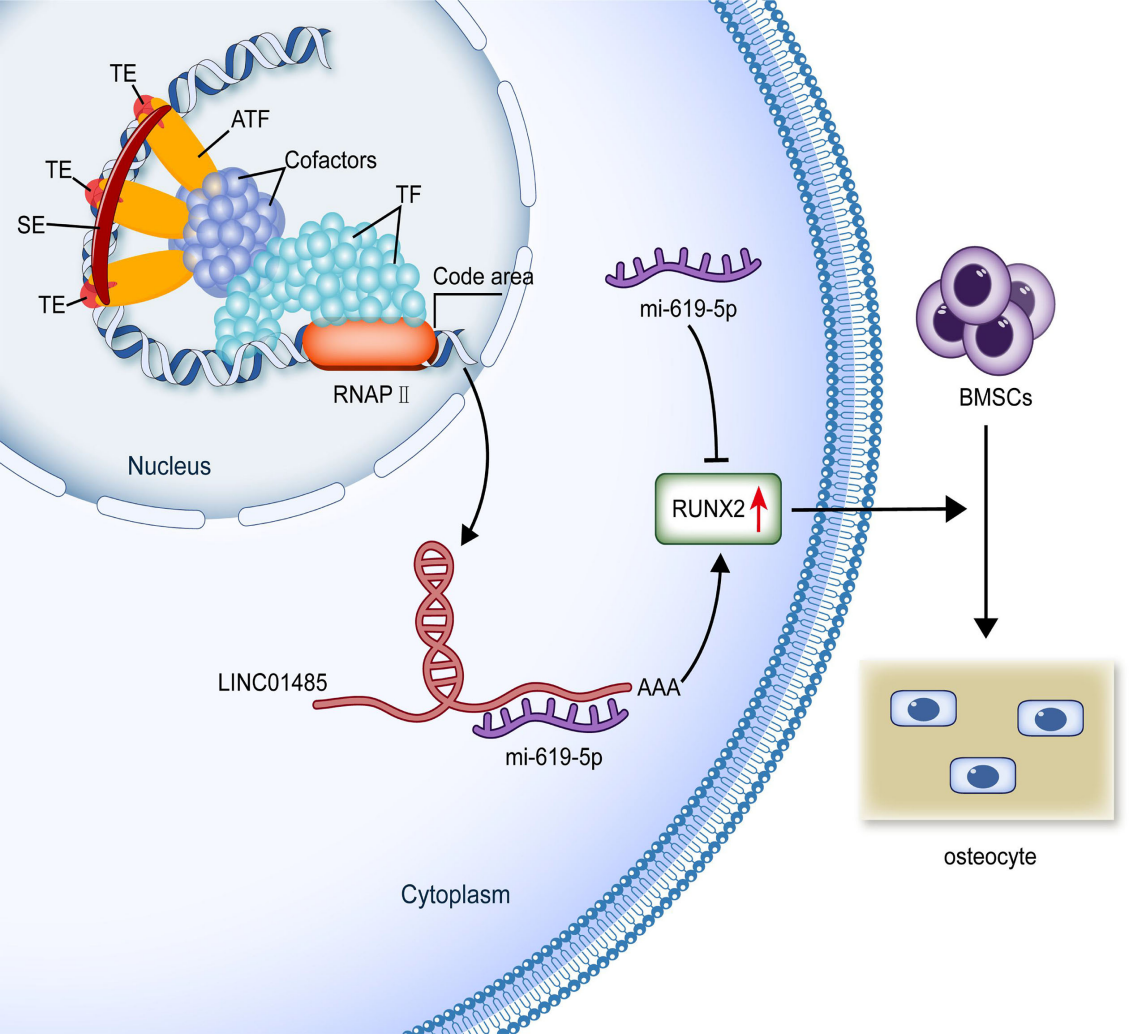
Production and possible functional mechanism of SE-lncRNA LINC01485. SEs are a genomic region composed of activity enhancer clusters enriched with many factors related to enhancer activity. SEs can increase the transcription and production of elncRNAs. SE-lncRNA LINC01485 competitively binds to miR-619-5p in the cytoplasm to promote RUNX2 expression regulating osteogenic differentiation of hBMSCs. (SE, super-enhancer; TE, typical enhancer; ATF, activating transcription factor; TF, transcription factor; RNAPII, RNA polymerase II; BMSCs, bone marrow mesenchymal stem cells.).

Earlier research has revealed the post-transcriptional interaction between lncRNA and miRNA as gene expression regulators in the process of BMSCs osteogenic differentiation (12). We predicted the mRNAs binding to miR-619-5p through bioinformatics. Among these genes, we focused on the osteogenic transcription factor RUNX2, based on the fact that Wnt signaling can promote osteogenesis by directly stimulating RUNX2 gene expression (62). RUNX2 is the primary regulating gene for the osteoblast phenotype (63) and binds to osteoblasts-specific cis-acting elements (OSE)-2 in the promoter region of osteogenic genes (64). RUNX2 has also been well demonstrated to promote the mineralization of bone nodules by up-regulating several osteoblast differentiation marker genes (e.g., ALP, COL1A1, OSX, OCN, and OPN) (65–67). These genes regulated by RUNX2 were found to be up-regulated when hBMSCs overexpressed LINC01485, and vice versa, these genes were downregulated upon interference with LINC01485. These results indirectly indicated that RUNX2 might be a target gene associated with LINC01485 during the osteodifferentiation process of hBMSCs. Previous studies have reported that the osteogenic promoting role of RUNX2 is regulated by other ceRNA signaling axes, including lncRNA NTF3-5/miR-93-3p/RUNX2 (68), LncRNA TUG1/miR-204-5p/RUNX2 (69), lncRNA MALAT1/miR-30/RUNX2 (17), lncRNA MALAT1/miR-124/RUNX2 (70), lncRNA MEG3/miR-140-5p/RUNX2 (71), lncRNA KCNQ1OT1/miR-138/RUNX2 (72), lncRNA MODR/miR-454/RUNX2 (73), and lncRNA DGCR5/miR-30d-5p/RUNX2 (74). Sponging of miRNAs by lncRNAs has been demonstrated as a regulatory mechanism in this context. By sponging miR-93-3p, lnc-NTF3-5 has been found to stimulate osteogenic differentiation of maxillary sinus membrane stem cells (68). LncRNA TUG1 is shown to promote osteogenic differentiation by up-regulating RUNX2 in aortic valve calcification through sponging miR-204-5p (69). Here, a negative relationship between miR-619-5p and LINC01485, a negative correlation between miR-619-5p and RUNX2, and a positive correlation between LINC01485 and RUNX2 were each demonstrated. In addition, the binding of miR-619-5p to RUNX2 was verified by luciferase assay. According to the competing endogenous RNA (ceRNA) hypothesis proposed by Salmena et al. (75), lncRNA can bind competitively to miRNAs and thus further enhance mRNA’s stability, transcription, and translation. We designed and conducted rescue experiments to verify the regulatory relationships among LINC01485, miR-619-5p, and RUNX2. Luciferase assays showed that miR-619-5p mimic inhibited the luciferase activity of RUNX2-WT, while the overexpression of LINC01485 promoted luciferase activity. At the cellular level, Western blot and ALP staining indicated that miR-619-5p inhibitor countered the effects of LINC01485 interference on RUNX2 and hBMSCs osteogenic differentiation. MiR-619-5p mimic also reduced the enhanced ALP staining effect of LINC01485 overexpression. These findings are consistent with the ceRNAs hypothesis, confirming the existence of endogenous sponge competition between LINC01485 and miR-619-5p, highlighting the role of the ceRNAs axis LINC01485/miR-619-5p/RUNX2 in regulating bone cell differentiation (**Figure 7**).

The potential limitations of current research must be considered. First, this study did not investigate osteogenesis by performing *in vivo* experiments to verify the reported *in vitro* findings. Animal experiments to confirm whether LNC01485 promotes osteogenesis by competitively binding to miR-619-5p are thus warranted. Second, the regulatory relationships involving SEs and lncRNAs in MSCs osteogenic differentiation remain to be addressed. The interaction between LINC01485 and SEs merits deeper exploration in future research. At the same time, the main findings bear potential implications for future research. The LINC01485/miR-619-5p/RUNX2 signaling axis revealed by the current study suggests a novel target for the genetic and epigenetic modification of hBMSCs. Such an approach could potentially enhance bone formation and thus hold promise in the stem cell-based bone tissue engineering area.

## Conclusion

In conclusion, this study identified that the SE-lncRNA LINC01485 was up-regulated during the osteogenic differentiation of hBMSCs. The upregulation of LINC015485 was found to promote osteogenic differentiation of hBMSCs by competitively binding miR-619-5p and up-regulating RUNX2 expression. The LINC01485/miR-619-5p/RUNX2 signaling axis emerged as a promising target for translation in the bone tissue engineering arena.

## Data Availability Statement

The original contributions presented in the study are included in the article/**Supplementary Material**. Further inquiries can be directed to the corresponding authors.

## Author Contributions

WG conceptualized the study design, carried out the experiments, performed the statistical analyses, and wrote the manuscript. XJ, WW, JL, ZM, HT, and SL were involved in the statistical analyses. HX and JZ supervised the whole research project. All authors edited and approved the final manuscript.

## Funding

This study was supported by the National Natural Science Foundation of China (No.81670950).

## Conflict of Interest

The authors declare that the research was conducted in the absence of any commercial or financial relationships that could be construed as a potential conflict of interest.

## Publisher’s Note

All claims expressed in this article are solely those of the authors and do not necessarily represent those of their affiliated organizations, or those of the publisher, the editors and the reviewers. Any product that may be evaluated in this article, or claim that may be made by its manufacturer, is not guaranteed or endorsed by the publisher.

## Supplementary Material

Supplementary Table 1 lncRNAs in the D14 group of specific SE-associated genes.

Supplementary Table 2 The forward and reverse sequence of primers for SE-associated lncRNAs in qRT-PCR assay.

Supplementary Table 3 The forward and reverse sequence of primers for genes examined in qRT-PCR assay.

Supplementary Table 4 LINC01485 interference sequence.

Supplementary Table 5 miR-619-5p mimic and miR-619-5p inhibitor sequence.

Supplementary Table 6 LINC01485 FISH probe sequence (FAM).

Supplementary Table 7 LINC01485 RAP probe sequence.

Supplementary Figure 1 The qRT-PCR validation of lncRNAs in the D14 group of specific SE-associated genes before and after osteogenic differentiation of hBMSCs.

Supplementary Figure 2 Electrophoretic diagram of RAP qRT-PCR products.

## Abbreviations

hBMSCs, human bone marrow mesenchymal stem cells; MSCs, marrow stem cells; lncRNAs, long non-coding RNAs; miRNAs, microRNAs; ceRNA, competitive endogenous RNA; RBP, RNA binding protein; SEs, super-enhancers; TEs, typical enhancers; Med1, Mediator1; RNAPII, RNA polymerase II; eRNAs/elncRNAs, enhancer-associated ncRNAs; SNP, single-nucleotide polymorphisms; AS, ankylosing spondylitis; SASEs, ankylosing spondylitis SNP-adjacent SEs; ASMSCs, MSCs from AS patients; SE-lncRNAs, super-enhancer-associated lncRNAs; mESCs, embryonic stem cells; EOC, epithelial ovarian cancer; CPCs, cardiac precursor cells; ChIP-seq, chromatin immunoprecipitation-sequencing; qRT-PCR, quantitative real-time polymerase chain reaction; TSS, transcription start site; FBS, fetal bovine serum; PVDF, polyvinylidene fluoride; ALP, alkaline phosphatase; PFA, paraformaldehyde; ARS, Alizarin Red S; FISH, fluorescence *in situ* hybridization; RAP, RNA antisense purification; DAPI, 4,6-diamidino-2-phenylindole; SD, standard deviation; RUNX2, runt-related transcription factor 2; OSX, Osterix; COL1A1, collagen type 1 alpha 1; OPN, osteopontin; OCN, osteocalcin; CDK, cyclin-dependent kinase; OSE, osteoblasts-specific cis-acting elements.
