# Supplementary material for: Super-Enhancer-Associated Long Non-Coding RNA LINC01485 Promotes Osteogenic Differentiation of Human Bone Marrow Mesenchymal Stem Cells by Regulating MiR-619-5p/RUNX2 Axis
Source: Front Endocrinol (Lausanne). 2022 May 19;13:846154. doi: 10.3389/fendo.2022.846154 (PMC9161675; doi:10.3389/fendo.2022.846154)
Supplement: Supplementary Table 1 — lncRNAs in the D14 group of specific SE-associated genes. [file DataSheet_1.docx]

**Supplementary Materials(tables,figures)**

**Supplementary tables**

**Table S1.** lncRNAs in the D14 group of specific SE-associated genes.

| **PeakID (cmd=annotatePeaks.pl 1.bedtools/osteoblast_only_SE.bed hg38)** | **Chr** | **Start** | **End** | **Strand** | **Peak Score** | **Focus Ratio/Region Size** | **Annotation** | **Detailed Annotation** | **Distance to TSS** | **Nearest PromoterID** | **Entrez ID** | **Nearest Unigene** | **Nearest Refseq** | **Nearest Ensembl** | **Gene Name** | **Gene Alias** | **Gene Description** | **Gene Type** |
| --- | --- | --- | --- | --- | --- | --- | --- | --- | --- | --- | --- | --- | --- | --- | --- | --- | --- | --- |
| 2_osteoblast_peak_1318_lociStitched | chr10 | 90674066 | 90676304 | + | 342 | NA | Intergenic | Intergenic | -134380 | NR_110657 | 101926942 | Hs.382666 | NR_110657 | ENSG00000236373 | LOC101926942 | - | uncharacterized LOC101926942 | ncRNA |
| 3_osteoblast_peak_10175_lociStitched | chr9 | 116542919 | 116549200 | + | 300 | NA | non-coding (NR_033973, exon 5 of 12) | non-coding (NR_033973, exon 5 of 12) | 41776 | NR_033973 | 100128505 | Hs.675821 | NR_033973 | ENSG00000229105 | ASTN2-AS1 | - | ASTN2 antisense RNA 1 | ncRNA |
| 2_osteoblast_peak_1263_lociStitched | chr10 | 76558351 | 76560508 | + | 282 | NA | Intergenic | Intergenic | -328615 | NR_120655 | 101929328 | Hs.664552 | NR_120655 | ENSG00000236467 | KCNMA1-AS1 | - | KCNMA1 antisense RNA 1 | ncRNA |
| 2_osteoblast_peak_1039_lociStitched | chr10 | 19719701 | 19731455 | + | 262 | NA | intron (NR_120646, intron 1 of 4) | L1MB4\|LINE\|L1 | 2972 | NR_120646 | 101928834 | Hs.676482 | NR_120646 | ENSG00000233968 | LOC101928834 | - | uncharacterized LOC101928834 | ncRNA |
| 1_osteoblast_peak_7723_lociStitched | chr5 | 173780891 | 173783174 | + | 240 | NA | Intergenic | Intergenic | 8910 | NR_108028 | 101928154 | Hs.366725 | NR_108028 |  | LINC01485 | - | long intergenic non-protein coding RNA 1485 | ncRNA |

**Table S2.** The forward and reverse sequence of primers for SE-associated lncRNAs in qRT-PCR assay.

| **lncRNAs** | **Sequence (5’- 3’)** |
| --- | --- |
| LOC101926942-F | CAGAGGGCTTTAACCAGCCA |
| LOC101926942-R | GCCACCTGATCACTCCATGA |
| ASTN2-AS1-F | AAGTTGGCACACAGCACTCT |
| ASTN2-AS1-R | ATTTCTACCTCCCGCTTGCC |
| KCNMA1-AS1-F | ATGTTCTTCCCAACCTGCCAA |
| KCNMA1-AS1-R | CTCAAACACGAGCGGACCAG |
| LOC101928834-F | ACGCTACAACCATTGCTCCA |
| LOC101928834-R | ACTGGACACAAACTCCAAGCA |
| LINC01485-F | CCGCATGCCAATGTGAAACT |
| LINC01485-R | AAGACCCTCCTCAGCAAAGC |

**Table S3.** The forward and reverse sequence of primers for genes examined in qRT-PCR assay.

| **Gene names** | **sequence(5’- 3’)** |
| --- | --- |
| RUNX2-F | TGGTTACTGTCATGGCGGGTA |
| RUNX2-R | TCTCAGATCGTTGAACCTTGCTA |
| OPN-F | AGACCCTTCCAAGTAAGTCC |
| OPN-R | TCATCTACATCATCAGAGTCGT |
| COL1A1-F | GCCAAGACGAAGACATCCCA |
| COL1A1-R | GGCAGTTCTTGGTCTCGTCA |
| OSX-F | CCTCTGCGGGACTCAACAAC |
| OSX-R | AGCCCATTAGTGCTTGTAAAGG |
| OCN-F | GTGCAGCCTTTGTGTCCAAG |
| OCN-R | AACTCGTCACAGTCCGGATT |
| GAPDH-F | GCACCGTCAAGGCTGAGAAC |
| GAPDH-R | TGGTGAAGACGCCAGTGGA |

**Table S4.** LINC01485 interference sequence.

| **Gene names** | **Sequence** | |
| --- | --- | --- |
|  | **sense（5'-3'）** | **antisense（5'-3'）** |
| LINC01485-si(Hum) | GGAAGGACAAAGAGAGAUUGG | AAUCUCUCUUUGUCCUUCCAG |
| siRNA-NC | UUCUCCGAACGUGUCACGUTT | ACGUGACACGUUCGGAGAATT |

**Table S5.** miR-619-5p mimic and miR-619-5p inhibitor sequence.

| **Gene names** | **Sequence(5'-3')** |
| --- | --- |
| miR-619-5p mimic | sense:GCTGGGATTACAGGCATGAGCC |
|  | antisense:CGACCCTAATGTCCGTACTCGG |
| miR-619-5p inhibitor | AGTGCTCTCTCTCCCCACGCTGGAGA |

**Table S6.** LINC01485 FISH probe sequence（FAM）.

| **Gene name** | **Probe sequence(5’-3’)** |
| --- | --- |
| LINC01485 | TCAGCAAAGCCCGAAAGTGCTCTCTCTCCCCACGCTGGAGACCAGTGT |

>NR_108028.1 Homo sapiens long intergenic non-protein coding RNA 1485 (LINC01485), long non-coding RNA

GTTTTTCTCCCGGTGCAGGAAAACATGAAGAACGGGGAAAGTAGCTGCCGCATGCCAATGTGAAACTCCGAGGAGTCTAGCCATTCTGATCTGGCCTTCCAGGTCATTAGGAGGCTCTGCCATCGTGTGAGGAGGGAAAGCCTGCTGTCTGGAGAGCACACTGGTCTCCAGCGTGGGGAGAGAGAGCACTTTCGGGCTTTGCTGAGGAGGGTCTTCAGAAAAAAGAGTACTGCCAACAAGAAGGCCCAATCAAGATCCTCCCACAACAAGAGCAGATTGTGAGCGTGGCAAGCTCTATGCTGAGCACTGGAAGGACAAAGAGAGATTGGGCACAGACTTCTCACTGATCCAGGTGATGAAGAGCTCACATCTGAGAGGATGTGACCATGGATGACCCACCAGAATGTCATGGGATCCAGCTACAGCCAAGGAAGCACAGAGCACCGTGGGAGCGATTTATTGTGCTTGGGGATGGGGAAGGGGACATGGGAGGAGCCATATTGAGAAAGTAGCATGGGTACTTGGCTTCACAGAGTGTGGCAGCTTTCATCAGGAAGTGATGGTGGGGGAACAGCGTCTCTGGCGGAGGGAAGCAGCAGGTGCAGAGGTGCAGAGGTGTGAGGAGTCTGTCACATTCTGGGAAGGGAGTATGGTTGGGTGACAATGCAAGACAGAGAAAGCAAGGAGGGAAGGAGGGGCCTAGCCTGAGGAATAAAGAATAATAAAAGCAGGAAAACAATT

**Table S7.** LINC01485 RAP probe sequence.

| **Three probes of Lnc01485 RAP** | **Sequence(5'-3')** |
| --- | --- |
| Probe 1 | TCAGCAAAGCCCGAAAGTGCTCTCT |
| Probe 2 | TCTCCCCACGCTGGAGACCAGTGTG |
| Probe 3 | AGTGCTCTCTCTCCCCACGCTGGAGA |

**Supplementary figures**

**
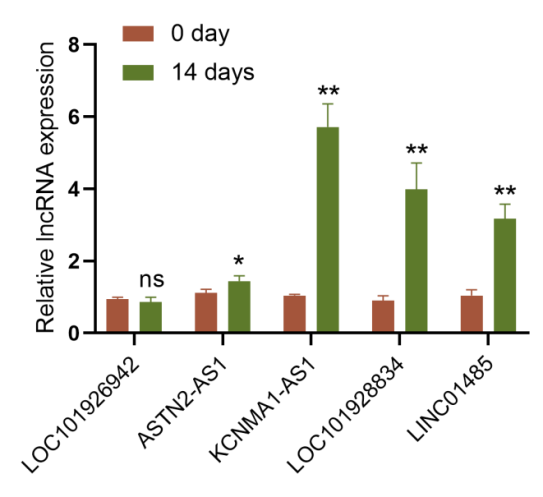
**

**Fig S1.** The qRT-PCR validation of lncRNAs in the D14 group of specific SE-associated genes before and after osteogenic differentiation of hBMSCs.


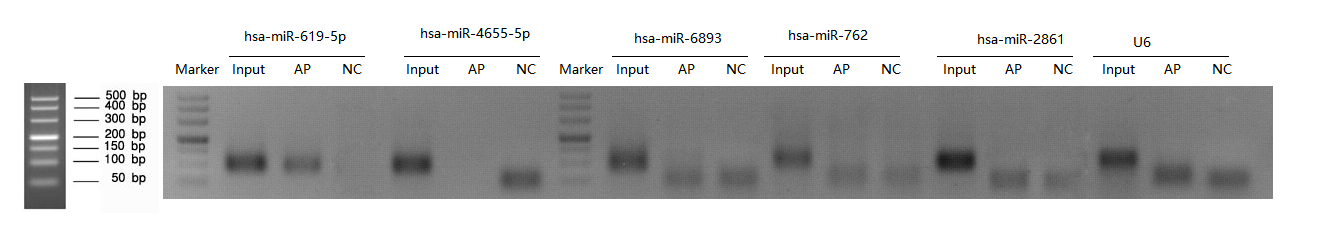


**Fig S2.** Electrophoretic diagram of RAP qRT-PCR products.
